# Undiagnosed Case of Klippel-Trenaunnay Syndrome Presenting as Extensive Heterotrophic Ossification and Flexion Deformity of Right Lower Limb Requiring Amputation: A Case Report

**DOI:** 10.31729/jnma.6932

**Published:** 2021-09-30

**Authors:** Kushal Gautam, Sangharsha Thapa, Anu Radha Twayana, Lokendra Chhantyal, Puskar Poudel, KC Avinash, Swati Chand

**Affiliations:** 1 Department of Paediatric Research, Patan Hospital, Lagankhel, Nepal; 2 Kathmandu University School of Medical Sciences, Dhulikhel, Kavrepalanchowk, Nepal; 3 Charak Memorial Hospital, Pokhara, Nepal; 4 Matri Shishu Hospital, Pokhara, Nepal; 5 Green Pastures Hospital, Pokhara, Nepal; 6 Department of Internal Medicine, Rochester General Hospital, Rochester, USA

**Keywords:** *flexion deformity*, *heterotrophic ossification*, *Klippel-Trenanunay syndrome*, *vascular malformation*

## Abstract

Klippel-Trenaunnay Syndrome is a rare disease characterized by a clinical triad of capillary malformation, soft tissue and bony hypertrophy, and atypical varicosity. This syndrome ranges from asymptomatic disease to life-threatening bleeding, embolism, and deformities. Management includes early diagnosis, prevention, and treatment of complications. We present a case of a 43-year-old male presenting with pain, swelling and deformity of the right leg for 30 years. On examination, diffusely enlarged tender right limb with several dark patchy discolorations, multiple tortuous vessels were found. Right leg X-ray showed heterotrophic ossification and distortion of ankle joint. Due to chronic severe pain, recurrent infection, contracture and flexion deformity of right leg, the patient underwent above knee amputation. This case focuses on the variable presentation and multiple problems faced by patients with Klippel-Trenaununay Syndrome as they get diagnosed late and shows the importance of high index of suspicion for early diagnosis and prevention of complications.

## INTRODUCTION

Klipper-Trenanunay syndrome (KTS) is a congenital disorder recognized at birth but can present in older children and adults.^1^ Its incidence is three to five cases per 100000 live births.^2^ It is sporadic. idiopathic, although familial cases are reported (autosomal dominant).^3-4^ Diagnosis requires two among three criteria.^1,5^ The vascular malformations are present from birth and affects the lower extremities.^6^ The rarity and complex presentation leads to wrong diagnosis, missed cases, increased morbidity, over investigation and treatment. Hence, physicians should have high index of suspicion. We present a case of complicated KTS in a 43 years man presenting with typical clinical features.

## CASE REPORT

A 43-year-old man presented with on and off pain, swelling and stiffness of the right lower limb for the past 30 years. The symptoms increased in severity since one month and affected his daily activities. The patient claimed to have few dark discolored patches in the right foot since birth. With advancing age, the discolored patch started to spread, and the entire left lower limb progressively increased in size with tortuous veins. He visited a nearby local clinic initially for his complaint, where he was treated with compression bandage. Over time, he started experiencing pain and developed ulcers in the skin surface. He visited a nearby health center where he was treated in line of cellulitis and filariasis. However his symptoms further worsened. In addition, he started developing deformity of right knee and hip joint, impairing his mobility, and warranting him to seek higher medical advice.

**Figure 1:**
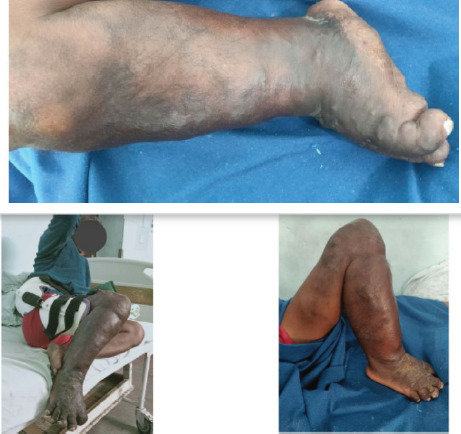
Swollen limb and dilated tortuous veins with limb hypertrophy.

On examination, diffusely enlarged and tender right limb with several dark patchy discolorations, multiple different sized tortuous vessels and feeble distal pulse were found (Figure 1).

There was fixed flexion deformity of the hip (30'), knee (90') and ankle (30' plantar-equinus) joints resulting in severe restriction of range of motion of the right lower limb. On the basis of medical history and clinical characteristics (triad of port wine stain, limb hypertrophy and lateral varicosity), provisional diagnosis of Klippel-Trenaunay Syndrome was made.

**Figure 2 A,B:**
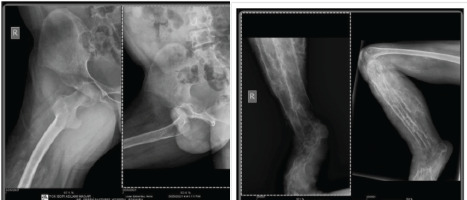
X-Ray (A) Right hip showing joint space reduction with patchy calcification (B) Knee and ankle showing osteopenia, soft tissue patchy calcification and distorted ankle joint.

X-ray of the right hip showed a concentric reduction of joint space with patchy calcifications and of the knee and ankle showed osteopenic bones, soft tissue patchy calcification, and a complete distortion of ankle joint (Figure 2 A,B).

The contralateral limb radiograph revealed a normal skeletal structure.

Doppler imaging of the right lower limb showed dilated torturous blood vessels in the right mid thigh and the medial leg perforators could be visualized. They were found to be normal in size with normal flow. Sapheno-femoral and sapheno-popliteal junctions were patent.

**Figure 3:**
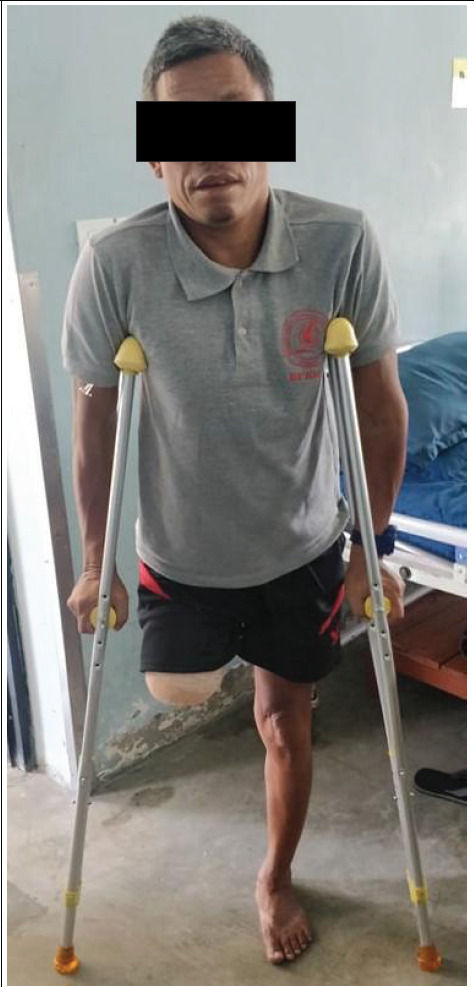
Post-amputation rehabilitation.

The patient was counseled about the disease, its severity, complications and possible need of amputation in view of chronic pain and poor functionality of the limb. He underwent above knee amputation of the deformed limb and psoas release to improve right hip joint flexion deformity. Following the procedure, the patient became pain free and had better mobility. He is now transferred to post amputation rehabilitation and is walking with the help of crutches (Figure 3).

Post amputation histopathological examination of the right thigh showed lobules of adipocytes hypertrophy skeletal muscle hypertrophy, venous thrombosis, and occlusion with large areas of calcification suggestive of KTS syndrome.

## DISCUSSION

Klippel-Trenaunay syndrome, named with two French physicians Klippel and Trenanunay, is a rare congenital disorder which is characterized by the presence of capillary malformations, venous malformation or varicosities and limb hypertrophy with or without lymphatic anomalies. The cutaneous manifestation of this syndrome is port wine stain, a vascular stain, usually noted at birth and most commonly involves the lower extremity. Venous malformations consists of dilated tortuous varicosities, a result of persistence of avalvular embryonal veins, usually in the lateral vein of thigh and sciatic vein.^7^ Limb hypertrophy is one of the common complain, noted from birth and is usually secondary to soft tissues and bony overgrowth. The affected limb can be larger as well as longer compared to the normal limb which can lead to limb length discrepancy and can lead to long term functional significance with compensatory tilting and imbalance of the pelvis resulting in abnormal gait, pain and functional limitations.^8^ It mostly affects single limb in around 80-85 percent of cases.^1,7^ The course of growth is usually unpredictable but can be progressive in nature. Although, we did not witness any other limb anomalies in our case but limb anomalies like macrodactyly, syndactyly, polydactyly and congenital hip dislocation have been reported.

A study was done in Mayo clinic to describe a series of 252 patients with KTS and capillary malformations (port wine stains) were found in 246 patients (98%), varicosities or venous malformations in 182 patients (72%) and limb hypertrophy in 170 (67%). All three features of KTS were present in 159 patients (63%), and 93 (37%) had two of the three features. Patients with at least two of the three cardinal features have been classified as having an incomplete form of KTS.^5^ Most cases of KTS are usually sporadic in origin although rare familial cases have been reported. Many genetic factors are identified as possible causative factors for KTS.^3,5^

Complications are related to the vascular pathologic process and ongoing clotting, most commonly a localized intravascular coagulopathy in areas of venous malformations and an increased risk for stasis dermatitis (due to chronic lymphedema and chronic venous insufficiency), thrombophlebitis, cellulitis with serious complications including thrombosis, coagulopathy, pulmonary embolism, congestive heart failure (in patients with arterio-venous malformations), and bleeding from abnormal vessels in the gut, kidney, and genitalia. Patients with large dilated and draining veins are prone to have pulmonary embolism. Varicosities and venous malformations over intrapelvic and intra-abdominal site can lead to occult or life threatening GI bleeding and haematuria.^9-11^ Despite these varieties of potential complication of KTS ,they occur in low rate. Fortunately, most of these complications are not found in the patient. But, the patient in our case had recurrent cellulitis with contracture of limbs leading to fixed flexion deformity and eventually a non functional limb.

Hence, clinical presentation of patients with KTS has a wide spectrum from incomplete, mild forms of port wine stains and few varicose veins causing only cosmetic deformity to severe disability associated with massive limb overgrowths, chronic pain syndrome, skin infections, arthritis, thromboembolism and life-threatening pelvic or recurrent rectal bleeding from venous malformations.

The diagnosis of KTS mainly depends upon the typical clinical characteristic features discussed above, which includes vascular stain, venous varicosities and limb overgrowth with or without limb malformations. Imaging tests in KTS should be focussed on evaluation of type, extent and severity of malformations.^1,12^ Doppler/duplex ultrasonography is helpful in detecting anomalies of the superficial and deep venous systems. It provides confirmation to establish patency, valvular incompetence, thrombosis and anatomical abnormalities such as atresia, hypoplasia or aneurysm.

Plain X-rays of the long bones are most helpful for the evaluation of leg length discrepancy, bony hypertrophy and additional osseous anomalies.^1^ MRI with or without gadolinium contrast is the imaging study of choice and is helpful in differentiating bone, fat, muscle hypertrophy and lymphedema. MRI also helps to define the nature and extent of vascular anomalies in patients with KTS.

Biopsy forroutinehistopathological(HPE) confirmations is usually not necessary to confirm the diagnosis of KTS. However, HPE has been done in our case and gross and microscopic features (venous malformation, lipomatous overgrowth) are suggestive of KTS.

The management of KTS has been largely conservative. Compression therapy, in the form of an elastic garment or compression bandage isthe cornerstone ofconservative management.^1^ This therapy is mostly indicated for chronic venous insufficiency, lymphedema, recurrent cellulitis and recurrent bleeding from capillary or venous malformations. Cellulitis and thrombophlebitis are usually managed with analgesics, elevation, antibiotics and corticosteroids. Local wound care, heel inserts, lifestyle modification, regular dressing with avoidance of specific drugs which can affect the clotting system may also be required to manage activities of daily living and improve the function of the limb.

Surgical intervention in the treatment of KTS remains controversial. Some studies suggest and consider surgery for either cosmetic deformity or pain relief, heaviness of the leg or infectious complication. Surgery is reserved selectively for symptomatic patients who are not candidates for less invasive treatment.^7^ Regarding limb hypertrophy, heel inserts are generally sufficient for limb discrepancies of 1.5 cm or less.^1,7^ Orthopaedic surgery may be needed for larger limb discrepancies. Amputation may be rarely needed when the large size of limb has interfered with daily activities of living.^7^ Amputation is also required due to recurrent infection, non healing ulcers or bleeding and to alleviate the chronic pain. Due to late presentation with complications like chronic pain, recurrent infection, contractures and fixed flexion deformity affecting daily life, he was not ideal candidate for conservative management. Hence, he was advised for above knee amputation and Psoas release of right lower limb.

Following the procedure, the patient became pain free and had better mobility. He is now transferred to post amputation rehabilitation and walking with the help of crutches.
